# Target product profile choices for intra-domiciliary malaria vector control pesticide products: repel or kill?

**DOI:** 10.1186/1475-2875-10-207

**Published:** 2011-07-28

**Authors:** Gerry F Killeen, Nakul Chitnis, Sarah J Moore, Fredros O Okumu

**Affiliations:** 1 Biomedical & Environmental Thematic Group, Ifakara Health Institute, PO Box 53, Ifakara, Kilombero District, Morogoro Region, Tanzania; 2 Vector Group, Liverpool School of Tropical Medicine, Pembroke Place, Liverpool L3 5QA, UK; 3 Department of Epidemiology and Public Health, Swiss Tropical and Public Health Institute, Basel, Switzerland; 4 University of Basel, Basel, Switzerland; 5 Department of Infectious Diseases, London School of Hygiene & Tropical Medicine, Keppel Street, London WC1E 7HT, UK

## Abstract

**Background:**

The most common pesticide products for controlling malaria-transmitting mosquitoes combine two distinct modes of action: 1) conventional insecticidal activity which kills mosquitoes exposed to the pesticide and 2) deterrence of mosquitoes away from protected humans. While deterrence enhances personal or household protection of long-lasting insecticidal nets and indoor residual sprays, it may also attenuate or even reverse communal protection if it diverts mosquitoes to non-users rather than killing them outright.

**Methods:**

A process-explicit model of malaria transmission is described which captures the sequential interaction between deterrent and toxic actions of vector control pesticides and accounts for the distinctive impacts of toxic activities which kill mosquitoes before or after they have fed upon the occupant of a covered house or sleeping space.

**Results:**

Increasing deterrency increases personal protection but consistently reduces communal protection because deterrent sub-lethal exposure inevitably reduces the proportion subsequently exposed to higher lethal doses. If the high coverage targets of the World Health Organization are achieved, purely toxic products with no deterrence are predicted to generally provide superior protection to non-users and even users, especially where vectors feed exclusively on humans and a substantial amount of transmission occurs outdoors. Remarkably, this is even the case if that product confers no personal protection and only kills mosquitoes after they have fed.

**Conclusions:**

Products with purely mosquito-toxic profiles may, therefore, be preferable for programmes with universal coverage targets, rather than those with equivalent toxicity but which also have higher deterrence. However, if purely mosquito-toxic products confer little personal protection because they do not deter mosquitoes and only kill them after they have fed, then they will require aggressive "catch up" campaigns, with behaviour change communication strategies that emphasize the communal nature of protection, to achieve high coverage rapidly.

## Background

The most important front line vector control strategies for malaria prevention rely on killing mosquitoes that enter human houses by delivering insecticidal products to these domestic targets in the form of indoor residual spray (IRS) or long-lasting insecticidal nets (LLINs) [1,2]. The common rationale underpinning these strategies is based on two well-established biological phenomena: 1) that the most important malaria vectors prefer to feed on humans and rest inside houses and 2) that a mosquito must feed several times on humans and, therefore repeatedly risk exposure to such insecticidal measures, before it is old enough to acquire, incubate and then transmit malaria to any human [3,4]. As the most common and important species of *Plasmodium *that cause human malaria infections are strict anthroponoses, malaria vectorial capacity of a given mosquito species is directly and closely related to its human-feeding propensity so these two phenomena obviously co-occur in the most important vector populations [5]. This is particularly true in sub-Saharan Africa where, with some interesting exceptions, the bulk of human exposure to *Anopheles gambiae *and *Anopheles funestus *has occurred inside houses and these species feed almost exlusively upon humans [6-8]. As a result, even coverage of only half of the human population with LLINs or IRS can deliver huge reductions of transmission and substantial alleviation of malaria burden in settings where the challenge of eliminating malaria is greatest [9,10]. Few public health interventions achieve such massive positive externality in the form of protecting those not directly covered [9-11] and the elegant way in which these measures exploit the biology of both the parasite and the vector is both intuitive and appealing [3,4,12]. The potential for community-level impact that is far greater than what can be achieved with personal protection alone is obviously hugely attractive [2,11,12], but this simple rationale and impressive recent progress with implementation masks a complex set of important product profile choices, which have thus far been made in the absence of decisive evidence or clear evaluation criteria.

However, the two most commonly used pesticides for controlling adult malaria vector mosquitoes, namely the synthetic pyrethroids and dichlorodiphenyltrichloroethane (DDT), combine two very distinct modes of action: 1) conventional toxicity which kills mosquitoes exposed to the pesticide while feeding or attempting to feed upon covered humans, 2) deterrence of mosquitoes away from those humans resulting from either irritation upon direct contact with the treated surface or even through spatial repellence from a distance of several meters [13-15]. Pyrethroids exhibit a strong combination of both contact irritant and spatial repellent properties, so that IRS and LLIN using these compounds often deter as many mosquitoes as they kill [16-20]. DDT is the only commonly used alternative to the pyrethroids for IRS and clearly has strong spatial repellency, as well as strong insecticidal effects upon mosquitoes that are not deterred and actually make contact [13,14].

While high levels of deterrence enhance the personal protection afforded by a pesticide product and, therefore, uptake by the public, it may also attenuate or even reverse communal protection [15] because it diverts mosquitoes to non-users [21] rather than killing them outright. Theoretical analysis suggests that where vectors have a strict preference for human hosts, or their preferred alternative hosts are absent, such deterrent properties may be counterproductive or even dangerous [15]. In principle, diversion of mosquitoes away from protected individuals might cancel out the community-level benefits to non-users arising from decreased mosquito survival and infection rates and could even result in increased exposure because bites are increasingly focused on the unprotected portion of the population [15].

Numerous large scale field trials of insecticidal nets or IRS have produced overwhelming encouraging results [9,10] but it is critical to note that these impacts result from products with a combination of deterrent and insecticidal properties. Even larger studies will be required to conclusively distinguish the community-level impacts of alternative profiles with deliberately and distinctly formulated toxic versus deterrent product profiles. It is therefore perhaps unsurprising that no such field trial has been conducted. While current guidelines for evaluating LLIN and IRS products in experimental huts [22] provide clear instructions on how to quantify personal protection and overall mortality rates of mosquitoes, it is not explicitly required to distinguish between toxic effects that kill mosquitoes before or after they feed and, with one exception [23], trials following these guidelines report only combined total mortality rates. Furthermore, consensus has yet to be attained regarding which of these evaluation criteria should be considered as primary and secondary or how the relative merits of these properties should be compared when evaluating existing products or designing new ones.

**Figure 1:**
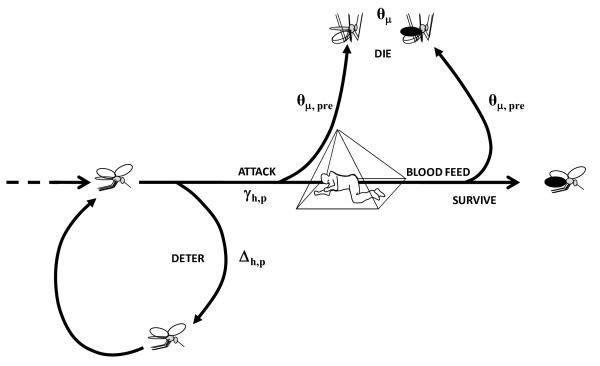
**A schematic outline how the model captures the sequential nature of deterrent (*θ_Δ_*) and toxic actions (*θ_Δ_*) of vector control pesticides and account for the distinctive impacts of toxic activities which kill mosquitoes before (*θ_μ,pre_*) or after (*θ_μ,post_*) they have fed upon the occupant of a covered house (IRS) or sleeping space (LLIN)**.

A process-based mathematical model of malaria transmission is outlined here, which captures the sequential interaction between deterrent and toxic actions of vector control pesticides and which accounts for the distinctive impacts of slow and fast-acting toxicity upon mosquitoes (Figure 1). This model is applied to explore how the interaction of deterrent and toxic actions affects both overall transmission intensity and its distribution across user and non-user groups in malarious communities. Furthermore, the consequent influence of alternative and hybrid product profiles upon the choice of optimal delivery system strategy is outlined and further potential applications for this model are discussed.

## Methods

Initially, a recently published deterministic model [24] was applied to elucidate how interactions between deterrent and insecticidal properties of hypothetical LLIN or IRS products might affect their impact upon malaria control when applied at high coverage across large populations. This exercise revealed that neither this formulation nor any of its predecessors [12,15,25] produced plausible, internally consistent outcomes for the probabilities of a mosquito attacking an encountered LLIN user and of successfully obtaining a blood meal when the proportion of human exposure that occurs at times when LLINs are used (*π_i_*) was set to values less than 1. The uncoupling of the impacts of *π_i _*upon repellence and insecticidal activity became particularly obvious when the hypothetical LLIN was defined as being 100% repellent (*θ_Δ _*= 1) and 100% insecticidal (*θ_μ _*= 1): such simulations indicated that mosquitoes were directly killed by these nets, despite the expectation that coupled and complete repellency should prevent any such fatal contact. Furthermore, this implausible exposure of mosquitoes to direct mortality risk despite complete diversion away from such hazard increased as the proportion of exposure the LLIN can potentially prevent (*π_i_*) decreased. Close examination of equations 6 and 7 of the original formulation [24] reveals how the previous approach caused the uncoupling of this conditionality to produce increasingly unrealistic outcomes as the fraction of exposure of indoor interventions for which the repellency does not apply (1- *π_i_*) increases, namely increasing estimated exposure of mosquitoes to the insecticidal activity and consequently nonsensically increasing insecticide-related mortality.

These flaws arise from inconsistent definition of protection, which was sometimes, but not always, considered to be synonymous with simply using a net. In simple terms, using a net is something that covered individuals only do for approximately one third of a typical day so protection must be assumed to be partial, even for the most nocturnal, indoor-biting vectors, regardless of net efficacy [25]. Such interactions between mosquito and human behaviours are best summarized for indoor interventions such as LLINs or IRS in terms of the proportion of human exposure that would otherwise occur indoors (*π_i_*) [25]. Published field estimates of this parameter for African malaria vector populations indicate that this proportion may fall far short of its optimal maximum value of 1 and may well be dropping in response to increasing selection pressure as ITN coverage increases [25-27]. Here these components of previously published formulations [12,15,24,25] are harmonized so that this increasingly important *de facto *gap in coverage is treated with far greater clarity and internal consistency (See Table 1 for parameter definitions). In the interests of brevity and simplicity of language, the model description below refers consistently to an LLIN product but relates equally to an IRS product. Here, the essential changes to the existing model are described in detail and a brief but comprehensive description of the overall model is provided.

**Table 1:** Definitions and explanations for symbols and abbreviations.

Symbol	Definition and explanation
*a*	Availability of individual hosts for attack: rate at which a single mosquito encounters and then attacks a given single host or pseudo-host [24].
*A*	Total availability of hosts and pseudo hosts: rate at which a single mosquito encounters and attacks all hosts and pseudo hosts [24].
*b_h_*	The mean number of bites upon humans per emerging mosquito during its lifetime [15,30].
*b*	The mean number of bites upon all human and non-human hosts per emerging mosquito during its lifetime.
*β_h_*	The mean number of infectious, sporozoite-infected bites upon humans per emerging mosquito during its lifetime [15,30].
*β*	The mean number of sporozoite-infected bites upon all hosts, regardless of their susceptibility to infection, per emerging mosquito during its lifetime.
*c*	Cattle [12,15,24,28,42].
*C_h_*	Crude coverage [12,15,24,28,42]: Proportion of people using LLIN as estimated in standardized malaria indicator surveys [82,83].
*C_h,p_*	Protective coverage: The proportion of all exposure of the human population which is effectively covered by LLIN use at times when that exposure actually occurs.
*DDT*	Dichloro-diphenyl-dicloroethylene [14].
*Δ*	Probability that a mosquito which encounters a host will be diverted from that host [12,15,24].
*ε*	Host-encounter rate: rate at which a single host-seeking mosquito encounters a given single hosts [12,15,24,28,42].
*E*	Emergence rate of mosquito vectors per year [12,15,24,28].
*EIR*	Entomological inoculation rate (mean number of infectious bites that an average individual human receives per year) [84-87].
*ϕ*	Probability that a mosquito which attacks a host will successfully feed upon that host [12,15,24,28,42].
*f*	Feeding cycle length: measured as the number of days it takes a single mosquito to get from one blood feed to the next [12,15,24,28].
*g*	Gestation interval: number of days a mosquito takes to digest a blood meal and return to searching for oviposition site [12,15,24,28].
*h *or *c*	Humans or cattle, respectively [12,15,24,28,42].
*IRS*	Indoor residual spraying [10,49]
*κ*	Human infectiousness to mosquitoes: probability of a vector becoming infected per human bite [29,30,88,89].
*LLIN*	Long-lasting insecticidal net [90]
*λ*	Relative availability for attack of a given non-human host type, calculated as quotient of the mean individual attack availability of those hosts divided by the mean individual attack availability of humans not using LLINs [24].
*μ*	Probability that a mosquito which attacks a host will die during the attack [12,15,24].
*η_o_*	Oviposition site-seeking interval: number of days a mosquito takes to find an oviposition site once it starts searching for it [12,15,24,28].
*η_v_*	Host-seeking interval: number of days a mosquito takes to find and attack a vertebrate host [12,15,24,28].
*net *or 0	LLIN user or non-user, respectively
*N*	Number of hosts [12,15,24,28].
*θ*_Δ_	Excess proportion of mosquitoes which are diverted while attempting to attack a human while using an LLIN [24].
*θ_μ_*	Excess proportion of mosquitoes which are killed while attacking a human while that person is using an LLIN [24].
*θ_μ,pre_*	Excess proportion of mosquitoes which are killed before blood feeding while attacking a human while using an LLIN.
*θ_μ,post_*	Excess proportion of mosquitoes which are killed after blood feeding while attacking a human while that person is using an LLIN.
*Ω *or 0	Intervention package scenarios consisting of a specific coverage with LLINs with specific deterrent and toxic properties, with 0 denoting baseline conditions with negligible net coverage, simulated by setting *C_h _*= 0.001 [24].
*π_i_*	The proportion of normal exposure to mosquito bites upon humans lacking LLINs, which occurs indoors at times when nets would normally be in use [25-27,37].
*p *or *u*	Specifies values of parameters for humans while actually using and protected by an LLIN, or those which are unprotected who do not use or are outside of their nets, respectively.
*P*	Probability that a resting mosquito survives any one day [15,91].
*P_f_*	Probability that a mosquito survives a single complete feeding cycle [12,15,24,28,30].
*P_ov_*	Probability that a mosquito survives any full day of the oviposition site-seeking interval or host-seeking interval [12,15,24].
*Q_h_*	Human blood index: the proportion of all blood meals from all hosts which are obtained from humans [12,15,24,28,30].
*γ*	Probability that a mosquito attacks an encountered host [12,15,24].
*ψ*	Relative exposure of different hosts other than unprotected humans to infectious mosquito bites: calculated as a ratio of exposure of those hosts to exposure of humans not using nets [24].
*WHO*	World Health Organization
*z*	Availability of blood from an individual host: rate at which a single mosquito encounters, attacks and successfully feeds upon a given single host [24]
*Z,Z_h_,Z_c_*	Total availability of blood from all hosts, all humans and all cattle, respectively: rate at which a single mosquito encounters, attacks and successfully feeds upon these host sets [24]
*Z_a_*	Total availability of aquatic habitats: rate at which a single mosquito encounters and successfully oviposits into all aquatic habitats

### Coverage, protection and host availability to mosquitoes

Protection is defined as being conditional upon both using a net and, more specifically, using a net at times when transmission occurs [25]. The *de facto *protective coverage of humans (*C_h,p_*) is therefore defined as being the product of crude coverage (*C_h_*) and the proportion of human exposure that occurs indoors while asleep at times when LLINs are used (*π_i_*) [25].(1)

The total availability for attack by mosquitoes [24] of protected (*A_h,p_*) and unprotected humans (*A_h,u_*) in the community is redefined so that individual users of nets exposed at times when they do not use them are considered to be unprotected. Thus, the effect of *π_i _*upon host availability is applied as a conditional probability that affects population-level parameters in a coupled manner, rather than a probability which is independently applied to each of distinct individual-scale processes it influences in an uncoupled manner. The total availability of hosts protected against attack by using a net is therefore adjusted for this fraction of exposure which is directly preventable (*π_i_*): The availability for attack of net users at times when those nets are used and therefore protect them is calculated as follows:(2)

Where a_h,p _is the availability for attack of an individual protected human, *N_h _*is the number of humans and C_h _is the crude coverage, estimated as the reported nightly usage rate.

The availability of the remaining fraction of humans which are unprotected (*A*_*h,u*_) because either they do not use a net (*A*_*h*,0,*u*_) or because they are exposed during times when the net is not used (*A_h,net,u_*) can be calculated as follows where *a_h,u _*is the attack availability of an unprotected individual.(3)

Which can also be expressed simply as follows in manner consistent with equation 2:(4)

Similarly, to estimate the total availability of blood (*Z*) from these same categories of human hosts, equivalent formulae based on the availability of blood from individual protected (*z_h,p_*) and unprotected (*z_h,u_*) human hosts are applied:(5)(6)(7)

By redefining protection and thus allowing for attenuated reductions of impact of insecticidal protection by human behaviours [25] at this population level the consistency and simplicity of parameters describing individual-level processes is improved. Individual mean (*a_h,p _*and *z_h,p_*) and population total availability parameters (*A_h,p _*and *Z_h,p_*) of the model are specified and calculated separately for protect and unprotected users and derived directly from the simpler respective un-weighted terms *γ_h,p _*and *ϕ_h,p _*, respectively. For diversion, this is achieved directly, similar to some previous formulations [12]:(8)

Where Δ*_h,p _*is the probability that a mosquito will divert away from an encountered, protected human host. However, the probability of feeding is expressed more explicitly than before, to consider only mortality which occurs before the mosquito feeds (*μ_h,p,pre_*) rather than total mortality (*μ_h,p_*) including those which feed but die soon afterwards:(9)

Where *μ_h,p,pre _*is the probability that a mosquito will die before feeding if it attacks a protected host.

These terms are calculated as follows based on the probabilities of diversion (Δ*_h,u_*) and death (*μ_h,u_*) for unprotected humans, combined with the additional probability of diversion (*θ*_Δ_) and death before feeding (*θ_μ,pre_*) caused by the deterrent and insecticidal properties of the net:(10)(11)

This distinction, between toxic activities that act fast enough to prevent blood feeding and those that do not, necessitates that the total excess attack-related mosquito mortality resulting from using an LLIN (*θ_μ_*) is specified as the sum of the excess mortality which occurs before (*θ_μ,pre_*) or after (*θ_μ,post_*) obtaining a blood meal:(12)

While insecticide-related mosquito mortality occurring after the mosquito has fed on the protected host does not contribute to personal protection, it does contribute to community-level suppression of malaria transmission by reducing population mean mosquito survival. The term *μ_h,p _*is therefore calculated separately as follows:(13)

This distinction between killing mosquitoes before or after feeding on the protected host allows the proportion of blood meals derived from humans (Q_h_) to be calculated as previously described [24] based on this revised feeding probability term. Note, however, that this parameter therefore includes fatal blood meals obtained from insecticide-protected humans which mosquitoes never live long enough to digest. The meaning of parameters depending on the availabilities various categories of attackable hosts (A), rather than blood sources *per se *(Z) described above, such as the duration of the host-seeking interval (η_ov_) and the probability of surviving host attack per feeding cycle (P_γ_) [24] are unaffected. Note also that, as described below in equation 14, the latter logically remains based on μ_h,p _rather than the new μ_h,p,pre _term.

### Implications of redefining coverage, protection and host availability for mosquito population parameters

Previous versions of this model incorporated the lack of an effect of an LLIN on outdoor malaria transmission *π_i _*by either treating it as a weighting term for calculating population mean values for feeding probability and attack-related mortality [12,25] or by applying directly to the individual level diversion and mortality processes [15,24]. The changed manner in which protection, coverage and availability are conceptually distributed (equations 1 to 7 and associated text), means that population-level parameters such as the proportion of blood meals obtained from humans (*Q_h_*) and mean host-seeking interval (*η_v_*), can all be simply calculated in terms of total host attack (*A*) and blood (*Z*) availability parameters exactly as previously described [15,24]. Note, however, that this means that the published breakdowns of these population-level parameters into functions of the products of numbers of hosts (*N*) and mean individual availabilities (*a *and *z*, respectively) [15,24] are no longer valid.

For other population-level parameters, simpler, more direct and intuitively satisfying derivations are implied. For example, this approach allows ready estimation of the probability of surviving host attack per feeding cycle (*P_γ_*) based on the mosquito mortality rates (μ) and corresponding community-wide total attack availabilities (A) of protected humans (h,p), unprotected humans (h,u) and cattle (c).(14)

Otherwise, all the mosquito population parameters are calculated exactly as previously described, and outlined as follows.

The mean seeking interval for vertebrate hosts (*η_v_*) can be calculated as the reciprocal of total host availability (*A*), using estimates of these feeding probabilities and their corresponding encounter rates [24,28]:(15)

The feeding cycle length (*g*) is calculated as the sum of the durations of the gestation period (*g*), the oviposition site-seeking interval (*η_v_*) and the vertebrate host-seeking interval (*η_v_*):(16)

Survival across all phases of the gonotrophic cycle is calculated as the distinct daily survival probability during each phase to the power of the respective time intervals, namely the host-seeking interval (*η_v_*), gestation period (*g*) and oviposition site-seeking interval (*η_o_*). The daily survival probability of a resting mosquito is defined as *P *and the survival probabilities during host-seeking and oviposition site-seeking are assumed to be equal and are both defined using the term *P_ov_*. The survival rate per feeding cycle (*P_f_*) was estimated as the combined probability that a vector survives gestation (*P^g^*), oviposition site-seeking , vertebrate host-seeking  and the eventual attack of a host :(17)

Similarly, the human blood index is calculated as the proportion of total blood availability accounted for by humans [24]:(18)

The biodemography component of the model is adapted to a daily cycle and cumulative survival up to each age (*x*) is estimated as follows [15]:(19)

Similarly, the sporozoite infection prevalence of mosquitoes at each age is considered in days, accounting for superinfection:(20)

where *κ *denotes the mean infectiousness of the human population to vector mosquitoes [29] and *n *is the duration of the sporogonic development period of the parasite from ingestion to infective sporozoite stages [30]. Survival and infectveness probabilities are calculated up to 40 days, after which the contributions of mosquitoes in these age classes to transmission become negligible. Note that *P_x _*is multiplied by *S_x _*to obtain the corresponding probability of being both alive and infective (*I_x_*) on each day

The following mosquito lifetime biodemographic parameters are calculated by summing these three age-specific outcomes as previously described [15,30]. The number of human bites the average mosquito takes in a lifetime (*b_h_*) is defined as the sum of the probabilities of surviving and feeding on a human at each age (*x*):(21)

Note that to enable incorporation of survival-dependent emergence rates, the number of human bites on all hosts, rather than just humans, per mosquito lifetime (*b*) is similarly calculated:(22)

Accounting for superinfection, the number of infectious bites on humans per mosquito lifetime (*β_h_*) is calculated as the product of the human blood index and sum of the products of the probabilities of biting and being infectious at each age [15,30]:(23)

Again, the number of sporozoite-infected bites on all hosts per mosquito lifetime (*β*), regardless of whether that host is susceptible to infection or not, is calculated similarly but ignoring the human blood index term:(24)

The overall sporozoite prevalence in the vector population (*S*) can then be calculated as *β_h _*divided by *b_h_*:(25)

### Epidemiological outcomes: dealing with partially covered, partially protected humans

Also, the entomologic inoculation rate (EIR) for non-users (*EIR*_*h*,0_) can be directly estimated based on the share of all available blood sources which a single non-user represents (*z_h,u_*/*Z*) multiplied by the total number of infectious bites on all hosts (*β *; equation 24) by all emerging mosquitoes (*E*):(26)

Alternatively, this parameter may be estimated by considering only infectious bites on human hosts (*β_h_*; equation 23) and therefore considering only the share of available human blood which such an individual represents:(27)

Nevertheless, it is essential to retain the protection-weighted mean terms for parameters which reflect the properties of individual net users who are only covered with the protective LLIN for proportion of their normal exposure (*π_i_*) and uncovered and unprotected for the remained (1 - *π_i_*). These terms are therefore retained but annotated more distinctly than previously [12] so that the attack probability (*γ_h,net _*rather than ) and feeding probability (*ϕ_h,net _*rather than ) reflect the mean of protected and unprotected periods for net users, but cannot be confused with the corresponding probabilities for net users during the specific periods when they are protected (*γ_h,p _*and *ϕ_h,p _*, respectively).(28)(29)

Consequently, derived terms such as attack availability (*a_net _*rather than ) and blood availability (*z_h,net _*rather than ), as well as corresponding terms for relative attack availability (*λ_h,net _*rather than *λ_h,p_*) and exposure to bites (*ψ_h,net _*rather than *ψ_h,p_*) compared with non-users, can be calculated as previously described.(30)(31)(32)(33)

Consequently, the EIR experienced by net users can be calculated by five different but consistent means:(34)

Additionally, the mean EIR experienced in scenario Ω by the mixture of net users and non-users which comprise the community (*ψ*_*h*,Ω_) can be independently calculated in three distinct ways which yield consistent results. Consistent with equation 22 of Okumu *et al. *[24], this parameter can be estimated by simply weighting the EIR parameters for net users and non-users according to crude coverage and the gap in coverage, respectively:(35)

However, it is also possible to calculate exactly the same values with a simpler formula derived from first principles, based on the assumptions of the very first of this family of models [30]:(36)

Reassuringly, identical values can also be calculated as described above by weighting the availability of blood from protected and unprotected individuals according to *de facto *protective coverage (*C_h,p_*) rather than crude coverage (*C_h_*).(37)

Similarly, the relative exposure of non-users and users of nets (*ψ*_*h*,0,Ω _and *ψ*_*h,net*,Ω _rather than *ψ*_*h,p*,Ω _and *ψ*_*h,p*,Ω_, respectively) and community-wide mean relative exposure (*ψ*_*h*,Ω_) in a given intervention scenario (Ω) is calculated exactly as previously described except that the terms *EIR*_*h*,0,0_, *EIR*_*h*,0,Ω _and *EIR*_*h*, *net*,Ω _replace *EIR*_*h*, *u*,0 _*EIR*_*h*, *u*,Ω _and *EIR*_*h*, *p*,Ω _to denote the EIR experienced by non-users in a scenario with no intervention and that of non-users and users under intervention scenario Ω, respectively:(38)(39)(40)

### Survival-dependent mosquito proliferation

Previous formulations of this model have assumed that larval habitats are always at their carrying capacity so the annual emergence rate of mosquitoes (*E*) is fixed, regardless of vector survival rates. In reality, vector populations experience dramatic seasonal fluctuations in larval habitat availability so while this assumption is largely true during drier times of the year when the quantity of habitat is static or contracting, it is rarely limiting during the onset or peak of the rains when vector populations can grow at their maximum reproduction rate [31,32]. Furthermore, observations of the differential impact of insecticide-treated nets upon sibling species composition within the *An. gambiae *complex [33,34] and impact of indoor-residual spraying upon inter-species competition within the *An. funestus *group [35,36], both confirm that oviposition input into larval habitats does limit vector population sizes. These simulations were, therefore, executed both with and without allowing for adult survival-dependent emergence rates which were calculated as follows.

Emergence rate was assumed to vary simply and linearly with mean number of successfully-completed feeding cycles by adult mosquitoes (*b*; Equation 22). Emergence rate in a given vector control scenario (*E*_Ω_) was therefore calculated as the product of the maximum emergence rate expected in the absence of any adult mosquito control (*E*_0_) and the relative value of the mean number of feeding cycles per mosquito lifetime in that scenario (*b*_Ω_), compared with such baseline conditions (*b*_0_):(41)

The calculations for the feeding cycle duration itself (*f*) as the sum of the gestation (*g*), oviposition site-seeking (*η_o_*) and vertebrate blood-seeking (*η_v_*) intervals are exactly as previously described [15]:(42)

Consistent with the previously published definition of host availability [24], it is assumed that protecting hosts does not alter their location, or the rate at which they are encountered by kinesis, but rather extends the spatial distribution of locations to which mosquitoes must disperse to in order to obtain blood. As hosts are increasingly protected, a greater mean number of hosts must be encountered before a blood meal can be successfully obtained. Longer host-seeking intervals, that include a greater mean number of unsuccessful host encounters, will inevitably result in a mean increase in the distance and duration of subsequent return journeys to oviposition sites. Calculation of the oviposition site-seeking interval (*η_o_*) is adapted to account for the expectation that mosquitoes forced to fly further and longer in search of blood will also have to fly proportionally further and longer in search of oviposition sites once the blood meal has been digested and eggs are matured. This term is calculated as the reciprocal of aquatic habitat availability, termed *Z_a _*rather than *A_a_*, as previously described [28], to maintain consistency with the separate definitions of rates of initiation and completion of resource utilization processes here and elsewhere [24]:(43)

However, here this term (*Z_a_*) is assumed to vary proportionally with vertebrate blood availability (*Z*) as it changes from baseline (0) to intervention (Ω) scenarios, reflecting the intrinsically endogenous relationship between host and aquatic habitat availability:(44)

### Parameterization of the model

The parameters of the model were set exactly as previously described [24] with the following adaptations, all of which are summarized in Table 2.

**Table 2:** Values and references for ecological parameters in the simulations

Definition	Symbol	Value	References
Total number of cattle	*N_c_*	1000	[15]
Total number of humans	*N_h_*	1000	[92]
Diversion probability from an unprotected vertebrate host (cattle or human)	*Δ_h,u_*	0.1	[93]
Mortality probability upon attacking an unprotected host	*μ_h,u_*	0.1	[93]
Mean availability of individual unprotected humans^a^	*a_h,u_*	1.2 × 10^-3^	[28,40]
Mean availability of individual cattle^b^	*a_c_*		
*An. arabiensis*		1.9 × 10^-3^	[28]
*An. gambiae s.s*.		2.5 × 10^-5^	[28,42]
Total availability of aquatic habitats	*Z_a_*	3	[28]
Duration of gestation	*g*	2	
Proportion of mosquitoes surviving per day while feeding while resting	*P*	0.9	[91]
Proportion of mosquitoes surviving per day while foraging for hosts or oviposition sites	*P_ov_*	0.85	Figure 2 and associated text
Duration of the parasite sporogonic development period	*n*	11	[30]
Human infectiousness to mosquitoes	*k*	0.03	[29]
Total number of adult mosquitoes emerging per year	*E*	2.0 × 107	[24]

^a ^The value of the parameter is equivalent to attacks per day per host-seeking vector perunprotected human.

^b ^The value of the parameter is equivalent to attacks per day per host-seeking vector per individual head of cattle and was different for the two vector species *Anopheles arabiensis *and *Anopheles gambiae sensu stricto*. With the exception of this parameter, all the other values are assumed to be identical for both species.

The term *π_i _*is set at a values of 0.90, consistent with published reports from areas with high coverage of untreated nets [25,37] and historical field observations for African vector populations from across Africa (Huho *et al*., Unpublished) or at 0.50, reflecting more recent observations from vector populations exposed to high coverage of LLINs, IRS or house screening [25-27,38].

**Figure 2:**
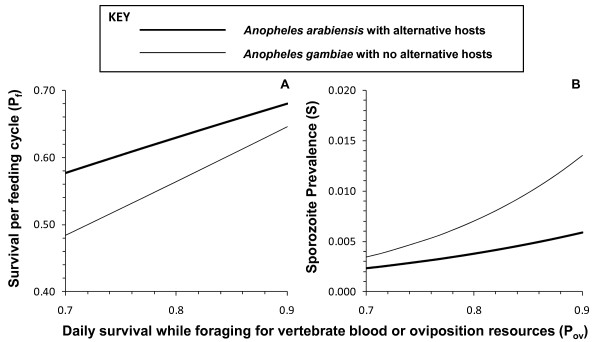
**The sensitivity of mosquito survival per feeding cycle (*P_f_*) and sporozoite infection prevalence (*S*) upon assumed values for daily survival while foraging for vertebrate blood or oviposition resources (*P_ov_*) in the absence of any LLIN or IRS intervention (*C_h _*= 0)**.

Previous modelling investigations [15,39] have illustrated that the eventual impact of deterrent pesticide products upon malaria transmission exposure for non-users is very sensitive to the assumed value for mosquito survival while foraging for vertebrate blood or oviposition site resources (*P_ov_*), parameter for which no field estimates exist to the authors knowledge. A range of values of were examined in the absence of any intervention measure (*C_h _*= 0) to determine an approximate value that is most compatible with the observed biodemographic profiles of real populations of vectors and sporogonic parasites in the field. Implausibly low values for the proportion of mosquitoes surviving each feeding cycle (*P_f_*) except at high assumed values of *P_ov_*, approaching the likely upper limit of 0.90 defined by the estimated survival rate of resting mosquitoes (Figure 2).

Furthermore, surprizingly low sporozoite prevalence (*S*) rates were predicted for both species, especially at the lower end of the range of assumed *P_ov_*, suggesting that values of the latter are high in nature. However, actual field estimates for survival per feeding cycle (*P_f _*= 0.62) and sporozoite prevalence (*S *= 0.016) for the village of Namwawala in the 1990s, where the crucial human population size (*N_h_*) and availability parameters (*a_h_*) were obtained from, were quite low by the standards African vector populations in the absence of LLINs or IRS and compare reasonably well with the *An. gambiae *scenario simulated here where *P_ov _*> 0.85. Note that although transmission in this village was dominated by *An. arabiensis *at this time, no significant cattle population existed so the *An. gambiae *scenario assuming no alternative hosts is most representative of this setting. While daily survival rates for actively foraging mosquitoes (*P_ov_*) must be somewhat lower than for resting mosquitoes, normal parity and sporozoite rates of African vector populations can only be plausibly explained if this difference is small, so *P_ov _*was set at 0.85 for all subsequent simulations.

All other parameter settings for the two vector population scenarios (*An. arabiensis *representing a mosquito that can exploit non-human hosts compared with *An. gambiae *which is almost exclusively dependent on humans for blood) are as previously described for a village with 1,000 people and an equal number of cattle [24].

Specifically, the mean individual attack availability of unprotected humans (*a_h,u_*) to *An. arabiensis *in this particular Tanzanian village in the 1990s was calculated as the reciprocal of the estimate of the mean vertebrate host-seeking interval (*η_v_*), based on the distribution of ovariolar stalks dilation status among host-seeking specimens [40], divided by the number of humans present at the time [24,28]. This approach to estimating this parameter was first described [28] before clear distinction between the availability of individual hosts for attack (*a_h,u_*) and the availability of individual blood sources *per se *(*z_h,u_*) had been explicitly outlined [24] but is even more appropriate when the former is specified. The same *a_h,u _*value of 1.2 × 10^-3 ^attacks per host per night per host-seeking mosquito was assumed for *An. gambiae*. The mean individual attack availability of cattle (*a_c_*) for each species was calculated by multiplying the equivalent parameter for humans (*a_h,u_*) by field estimates [41] of the relative availability of cattle blood, compared to that of humans (*ψ_c_*), for both vector species [42], yielding estimates of 1.9 × 10^-3 ^and 2.5 × 10^-5 ^attacks per host per night per host-seeking mosquito, for *An. arabiensis *and *An. gambiae*, respectively. Note that this calculation assumes that for unprotected hosts, the probability of successfully feeding upon an attacked host is equivalent for the two host types (*μ_h,u _*= *μ_c_*) so that the relative availability of cattle for attack is equivalent to the relative availability of cattle blood (*λ_c _*= *ψ_c_*).

Consistent with previous simulations, the maximum emergence rate of mosquitoes in the absence of adult mosquito control measures (*E*_0_) was set at 2 × 10^7 ^adult mosquitoes per year. Except where stated otherwise, crude coverage of humans was set at 80% (*C_h _*= 0.8) in line with the Roll Back Malaria targets for coverage of all age groups with LLINs which represents an ambitious but realistically achievable target for most malaria afflicted developing nations.

## Results

**Figure 3:**
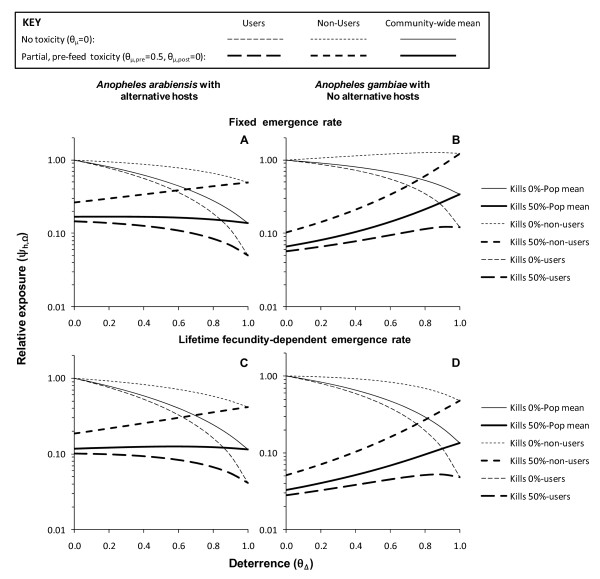
**Predicted impact of increasing levels of deterrence (*θ_Δ_*) upon exposure to malaria transmission for LLIN or IRS products with (*θ_μ,pre _*= 0.5, *θ_μ,post _*= 0 and without (*θ_μ,pre _*= 0, *θ_μ,post _*= 0) toxic properties, assuming either fixed or survival-dependent emergence rates (*E*) at 80% crude coverage (*C_h _*= 0.8)**.

The fundamental trade-off between toxic and deterrent actions (Figure 1) is clearly illustrated by the simulation results presented in Figure 3, all of which are based on the assumption that 80% of humans use LLINs (*C_h _*= 0.8). Predictions for toxic-deterrent hybrid product profiles (*θ_μ,pre _*= 0.5, *θ_μ,post _*= 0, *θ_Δ _*> 0) converge with those for purely deterrent product profiles (*θ_μ,pre _*= *θ_μ,post _*= 0, *θ_Δ _*> 0) once deterrence reaches 100% efficacy and prevents any fatal contact with the active ingredient (*θ_Δ _*= 1 so that γ*_h,p _= *0). This is to say that given maximum diversion, the probability that a mosquito would attack a protected host becomes zero. A number of further observations suggest this trade-off should be carefully considered when defining a target product profile for developing or selecting a malaria vector control pesticide formulation.

A partially efficacious but purely fast-acting toxic product ((*θ_μ,pre _*= 0.5, *θ_μ,post _*= 0, *θ_Δ _*> 0) consistently delivers better protection of non-users than a completely efficacious but purely deterrent ((*θ_μ,pre _*= 0, *θ_μ,post _*= 0, *θ_Δ _*= 1.0) product (Figure 3). A reasonable degree of community-level protection for non-users is accrued where attractive, non-human hosts exist for diverted mosquitoes to feed upon. However, in the absence of such alternative blood sources, the unprotected minority of the human population could experience greater exposure and this negative externality increases with increasing deterrence (Figure 3b). Furthermore, the consistently strong community-level benefits obtained by non-users when their neighbours use pesticide products with purely toxic activity profiles are undermined in all scenarios by supplementing these lethal effects with increasing levels of deterrence (Figure 3).

Where alternative animal hosts exist, benefits for users of toxic nets are modestly enhanced as high levels of personal protection provided by strong deterrent properties (*θ_Δ _*> 0.5) are realized (Figure 3). However, this results in an approximate break-even scenario, in terms of mean relative exposure across the entire community because increased benefit for users is offset by reduced benefit for non-users (Figure 3). Where alternative sources of blood are absent, increasing deterrence actually progressively undermines protection of users because the increased personal protection conferred is more than counterbalanced by dramatically attenuated community-level impact (Figure 3).

Note that for all of these conclusions, the model which includes survival-dependent emergence rates (Figure 3c and 3d versus 3a and 3b) improves the predicted outcomes for purely deterrent products and toxic-deterrent hybrids but in no case does so dramatically enough to alter the overall trend or conclusions reached (Figure 3). These simulations suggest that purely toxic products are preferable to purely deterrent ones and that enhancing the personal protection afforded by a toxic product by increasing its repellent or irritant properties will consistently undermine or even reverse communal protection of non-users. In fact, where vectors lack alternative non-human hosts, increasing deterrence may even undermine benefits for users because the degree of community-level protection obtained with purely toxic products is far greater than personal protection at the high levels of coverage now considered as healthy targets for any malaria control programme [1,2].

**Figure 4:**
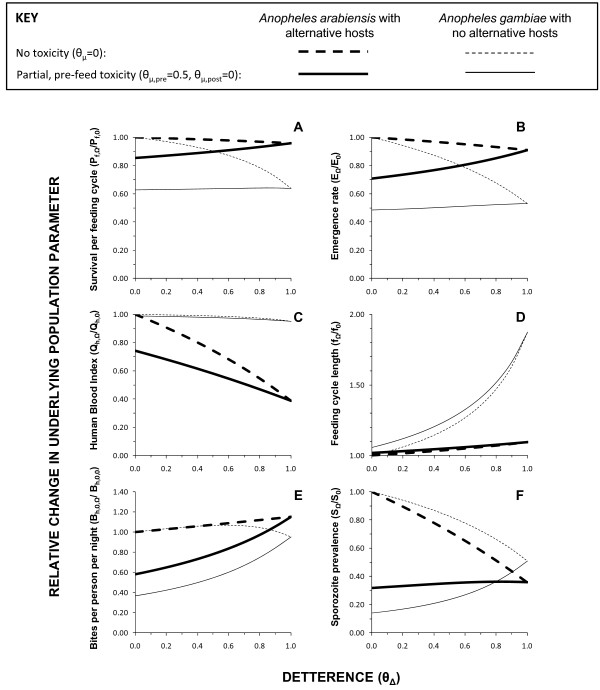
**Predicted impact of increasing levels of deterrence (*θ_Δ_*) upon underlying biodemographic mosquito and sprogonic-stage parasite population parameters that determine malaria transmission for LLIN or IRS products with (*θ_μ,pre _*= 0.5, *θ_μ,post _*= 0) and without (*θ_μ,pre _*= 0, *θ_μ,post _*= 0) toxic properties at 80% crude coverage (*C_h _*= 0.8)**. **Only the model with survival-dependent emergence rates (*E*) is presented.**

Figure 4 illustrates how such counterintuitive predictions may be rationalized by examining the underlying biodemographic parameters describing the vector and sporogonic-stage parasite populations, which ultimately determine impact on malaria transmission. Vector survival per feeding cycle (*P_f_*) is the most important single determinant of malaria transmission intensity other than temperature and is substantially reduced by toxic, deterrent and toxic-deterrent hybrid products where no alternative blood hosts exist (Figure 4a). Where alternative hosts occur, only toxic products with little or no deterrence are predicted to usefully reduce vector survival (*P_f_*). Regardless of whether alternative hosts are present, increasing deterrence of toxic products consistently weakens impact upon this most important target for adult malaria vector control, modest reductions of which result in quasi-exponential suppression of transmission [4,15,39]. As the impact upon vector reproduction (*E*) has been modelled as a linear function of the number of bites taken per lifetime (*b_h_*), itself a simple function of survival (*P_f_*) [15], it is unsurprising that the impact of these various product profiles mirrors that upon survival (Figure 4b). Being a squared term in all malaria transmission models [4,30,39], the proportion of blood meals that the vector population obtains from humans is the next most important determinant of malaria transmission intensity at global [43] and local level [15,39,42]. Where alternative sources of blood are available, deterrence can dramatically reduce this target parameter in its own right and also enhances the impact of toxic products when added as a supplementary activity (Figure 4c). In the absence of alternative hosts, no toxic, deterrent or hybrid product has any meaningful impact on this target parameter. Consistent with outputs of previous formulations [15], increasing deterrence can greatly extend the feeding cycle length (*f*) of the vector where no alternative non-human hosts exits but has a very modest effect where they are present (Figure 4d). Consistent with the recently revised, distinct definitions of host and blood availabilities [24], toxicity has no influence on this determinant of mosquito survival (*P_f_*), feeding frequency (1/*_f_*), reproduction (*E*) and transmission potential (*b_h_, S*).

In summary, toxic products consistently reduce vector survival and reproduction rates, especially in the absence of alternative blood sources. In places where such non-human preferred hosts exist, toxic products only reduce the proportion of blood meals that are human but have no impact on vector feeding cycle length. In contrast, purely deterrent products only have useful impacts upon the proportion of blood meals obtained from humans where alternative hosts exist and upon feeding cycles length where they are absent. Deterrent products, therefore, impact one of these two target parameters or the other and it is notable that neither has as strong an influence upon transmission as survival, particularly when further impact upon mosquito reproduction rates is considered.

**Figure 5:**
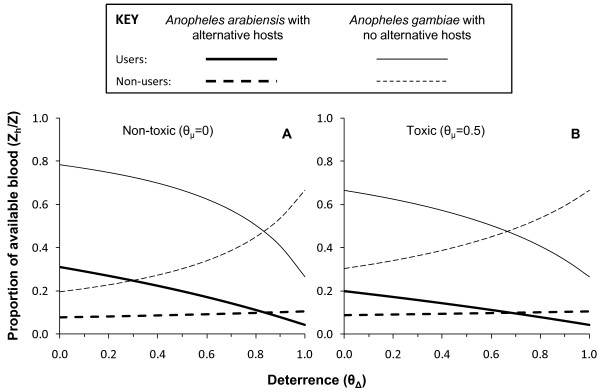
**Predicted impact of increasing levels of deterrence upon the share of total blood availability (*Z*) that human users and non-users of LLINs (*Z_h_*) constitute as the deterrence of an LLIN or IRS product at 80% crude coverage (*C_h _*= 0.8)**.

By definition (Figure 1), increasing deterrence of a product inevitably increases the proportion of available blood that non-users constitute at any given coverage level (Figure 5) and therefore the share of mosquito bites they experience, regardless of whether that product is toxic or not. When the predicted extent of this inequitable redistribution of biting mosquitoes (Figure 5) is combined with the predicted impacts upon the biodemographic properties of the vector population (Figure 4a to 4d), the overall impact is to increase biting rates for non-users (Figure 4e) even where alternative blood sources are absent so vector survival (Figure 4a) and reproduction (Figure 4b) are reduced because the availability of blood becomes limiting. This effect is so dramatic that, even for toxic products, increased exposure of non-users to bites can occur at high levels of deterrence (*θ_Δ _*> 0.8). While such negative externality in the form of diverting biting mosquitoes to unprotected non-users has been envisaged and discussed previously, the simulated impact of increasing deterrence of toxic products upon the sporozoite infection prevalence are perhaps more interesting. Consistent with previous simulations [15], purely deterrent products consistently reduce sporozoite prevalence (Figure 4f) by either lowering human blood indices where alternative hosts are available (Figure 4c) or by reducing survival (Figure 4a) and extending feeding cycle length (Figure 4d) where they are not. More surprising is the prediction that increasing the deterrence of a toxic product can attenuate impact upon sporozoite prevalence. In the case of vector populations lacking an alternative non-human host, this rebound of sporozoite infection prevalence arising from enhancing the personal protection provided by the product, by increasing irritant of repellent properties, is quite substantial. In fact this weakening of impact upon sporozoite prevalence may be as important a contributor to the dramatic attenuation of overall impact upon transmission (Figure 3b and 3d) as redistribution of bites to unprotected non-users (Figure 5).

**Figure 6:**
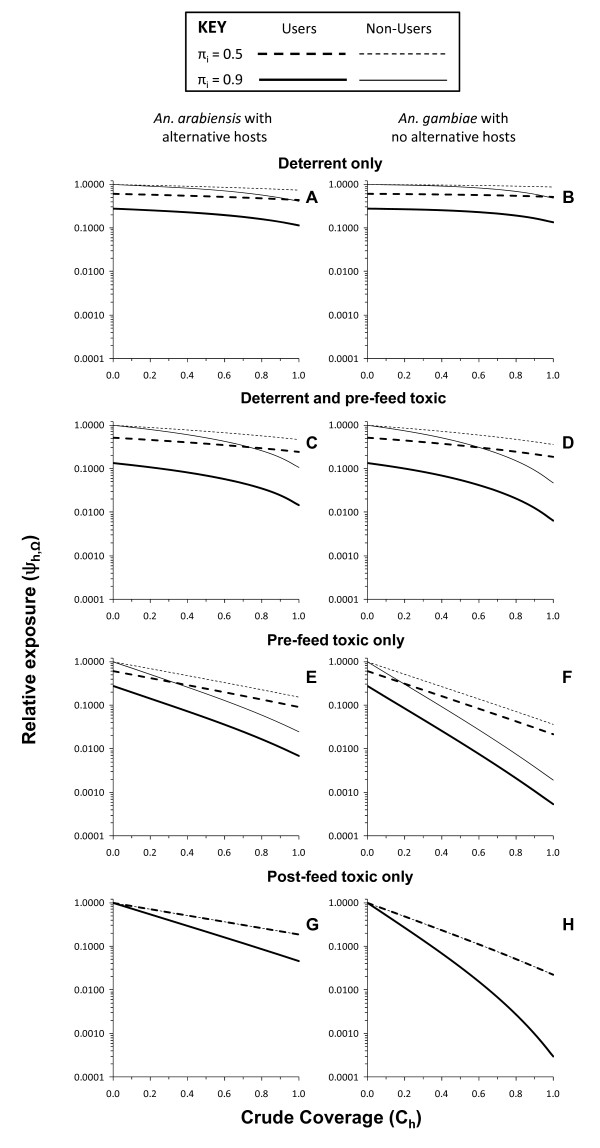
**Predicted impact of LLIN or IRS products with purely pre-feeding toxic (*θ_μ,pre _*= 0.8 *θ_μ,post _*= 0, *θ_Δ _*= 0), post-feeding toxic (*θ_μ,pre _*= 0, *θ_μ,post _*= 0.8, *θ_Δ _*= 0), deterrent (*θ_μ,pre _*= 0, *θ_μ,post _*= 0, *θ_Δ _*= 0.8) and pre-feeding toxic-deterrent hybrid (*θ_μ,pre _*= 0.8 *θ_μ,post _*= 0, *θ_Δ _*= 0.8) properties upon malaria transmission exposure for users and non-users where either most (*π_i _*= 0.9) or half (*π_i _*= 0.5) of baseline transmission occurs indoors**.

Figure 6 illustrates just how much more efficacious a purely toxic product can be. In both vector-host scenarios, toxic (Figure 6c and 6d) or toxic-deterrent hybrids (Figure 6e and 6f) are clearly superior to non-toxic deterrent products (Figure 6a and 6b). Obviously, the toxic but not deterrent product confers less personal protection than the toxic-deterrent hybrid but correspondingly provides the best communal protection for non-users as coverage increases. Even in the *Anopheles arabiensis *scenario where alternative hosts are available, the benefit to users of a purely toxic product arising from combined personal and community-level protection exceeds that of a toxic-deterrent hybrid at 57% coverage where baseline transmission primarily occurs indoors (*π_i _*= 0.9) and only 27% coverage where an equal amount of baseline transmission occurs outdoors (*π_i _*= 0.5). For *An. gambiae*-dominated transmission systems without alternative blood hosts, the advantage of purely toxic products conferring less protection than those supplemented with deterrence is even more dramatic and obvious, with almost three orders of reduction of transmission possible within feasible coverage targets and the purely toxic product providing greater protection than the hybrid at 22 and 12% coverage, respectively, where most (*π_i _*= 0.9) and half (*π_i _*= 0.5) of baseline transmission occurs indoors. Not only do purely toxic products have greater efficacy at reasonable coverage levels, they are also more robust to attenuation by outdoor-feeding behaviours in the target vector population (*π_i _*= 0.5) because, under such conditions, deterrent products simply divert mosquitoes to feeding on users at times when they are unprotected, especially when no alternative non-human hosts are available.

**Figure 7:**
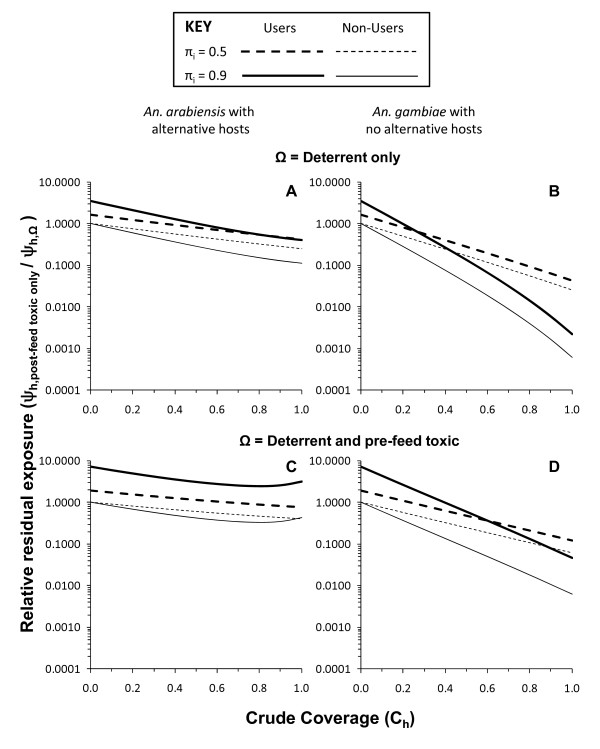
**Relative residual malaria transmission achieved with varying levels of crude coverage of a purely post-feeding toxic LLIN or IRS product (*θ_μ,pre _*= 0 *θ_μ,post _*= 0.8, *θ_Δ _*= 0), compared with products with both purely deterrent (*θ_μ,pre _*= 0, *θ_μ,post _*= 0, *θ_Δ _*= 0.8) and pre-feeding toxic-deterrent hybrid (*θ_μ,pre _*= 0.8 *θ_μ,post _*= 0, *θ_Δ _*= 0.8) properties for users and non-users where either most (*π_i _*= 0.9) or half (*π_i _*= 0.5) of baseline transmission occurs indoors**.

With the exception of the two bottom panels of Figure 6, all toxic actions simulated thus far are assumed to kill mosquitoes before they can bite the occupant of the house or net. This kind of scenario is best reflected in reality by LLINs with which the pyrethroid insecticide activity is specifically applied to a physical barrier between the attacking mosquito and the protected host so that most dead mosquitoes collected in experimental hut trials are unfed. However, in the case of IRS with non-deterrent insecticides, such as entomopathogenic fungi [44], bendiocarb [19], chlorpyrifos methyl [45], and even pyrethroid-based LLINs that have been depleted of insecticide after several years of use [16], most mosquitoes killed succeed in feeding before dying so little, if any, personal protection is conferred. Figure 6g and 6h represent such a scenario and this is reflected in the fact that the predicted degree of protection of users and non-users is identical because this is exclusively mediated by community-level suppression of transmission. Obviously, a purely insecticidal product which kills mosquitoes fast enough to prevent blood feeding and therefore also confers personal protection (Figure 6e and 6f) is preferable to one that kills them afterwards and does not (Figure 6g and 6h). Nevertheless, even a purely toxic product, which confers no personal protection because it only kills mosquitoes after they have fed (Figure 6g and 6h), is a consistently better option in terms of protection of non-users than products with deterrent properties, regardless of whether (Figure 6c and 6d) or not (Figure 6a and 6b) that product also has insecticidal activity that kills mosquitoes before feeding. Comparing the residual transmission levels achieved with products that confer only community-level protection through purely post-feeding toxicity with that attained by more conventional products with purely deterrent or deterrent plus pre-feeding insecticidal activities (Figure 7), shows that the non-user is always better off with the former.

For zoophagic vectors with alternative hosts available that predominantly feed indoors (*π_i _*= 0.9), deterrent plus pre-feeding insecticidal activity attains lower residual transmission for users than purely post-feeding insecticidal activity. However, such a scenario with most of the alternative hosts and mosquito feeding activity occurring indoors is probably unusual and occurs in a limited number of settings across the tropics. In all other scenarios, especially where half of transmission occurs outdoors (*π_i _*= 0.5), the purely post-feeding insecticide confers superior overall protection to users, despite complete lack of personal protection, once a minimum coverage threshold is surpassed. Compared with pure deterrents, overall protection of users becomes greater for the purely post-feeding insecticidal product at quite modest crude coverage levels (49 and 20% for *An. arabiensis *with alternative hosts and *An. gambiae *without them, respectively) where most transmission occurs indoors (*π_i _*= 0.9) and even lower thresholds (35 and 14%, respectively) where outdoor feeding and/or resting is common (*π_i _*= 0.5). Compared with products combining deterrent with pre-feeding insecticidal activity analogous to LLINs, similar patterns were observed, with the consistent disadvantage of purely post-feeding toxicity where alternative hosts exist and most transmission occurs indoors being reversed when outdoor transmission becomes important and crude coverage exceeds 65%, while it becomes consistently advantageous for vectors lacking alternative non-human hosts at remarkably low coverage thresholds of 39% for predominantly indoor transmission and only 22% where half of transmission occurs outdoors.

## Discussion

The idea that deterrency reduces the impact of toxic activities of pesticides upon mosquito survival is long-established [46] and was discussed extensively during the previous global campaign to eradicate malaria [47-49] as well as the beginning of the more recent drive to promote scale up of LLINs and IRS for control purposes [50]. Deliberate design of pesticide-based vector control products to match ideal target product profiles has recently been reprioritized as an important issue [13] now that more ambitious programmes to control, eliminate or even eradicate of malaria are back on the global agenda [51,52]. The process-explicit model of malaria transmission described here captures the sequential interaction between deterrent and toxic actions of vector control pesticides. In simple terms, it is not realistic to expect that one can discourage mosquitoes from making contact with an active ingredient without compromising the ability of that pesticide to kill them (Figure 1). Sub-lethal exposure that deters mosquitoes inevitably reduces the proportion which is subsequently exposed to higher, lethal doses. In fact, the extreme example outlined on the right hand side of all the panels in Figures 3 and 4, wherein the predicted impacts of products with and without toxic activities converge once 100% deterrency is achieved, clearly demonstrates that this is a choice which must be made: increasing deterrency and personal protection must always be traded off against reduced toxicity-mediated mosquito mortality and potent communal level protection where high coverage is achieved.

The assumptions and definitions of this model (Figure 1 and Methods) are also fully compatible with recent recommendations that toxic activities and both forms of deterrence, namely contact irritance and spatial repellence, are distinct and that each pesticide-affected mosquito collected in an experimental hut trial should be classified as having either responded in a manner characteristic of only one of these possible outcomes [13]. While parameter estimates from published studies have been deliberately avoided to minimize any appearance of recommending for or against specific commercial product choices, this model can be readily and directly parameterized from existing, standardized experimental hut evaluations. The diversion term *θ_Δ _*is estimated directly as the proportional change in the number of mosquitoes which either do not enter the hut (deterrence) or which leave unfed (excito-repellency) but do not subsequently die. The mortality terms *θ_μ,pre _*and *θ_μ,post _*are estimated as the increased proportion of all mosquitoes caught in a hut with a given LLIN or IRS product which were found dead or that subsequently died which were either unfed or fed respectively. However, to enable the application of this model to such experimental hut study outcomes, published summaries will need to explicitly distinguish between pre- and post-feeding mortality [23] and will ideally include the raw data as supplementary online material. The model described also accounts for the distinctive impacts of toxic activities, which kill mosquitoes before or after they have fed upon the occupant of a covered house or sleeping space. A variety of well-established domestic vector control products and emerging new technologies only kill mosquitoes after they have fed because they are applied as IRS formulations or because they are slow acting. Such alternatives to DDT or pyrethroids include entomopathogenic fungi [44], bendiocarb [19], chlorpyrifos methyl [45], and even pyrethroid-based LLINs that have been depleted of insecticide after several years of use [16], can take hours or days to kill most of the exposed mosquitoes but clearly can deliver massive levels of malaria control if sufficient coverage can be achieved. Only two previous models distinguish between the effects of pesticidal products that kill mosquitoes before and after they feed upon humans [14,53]. While one only considers processes that occur in houses and does not capture the community-level effects of different product profiles upon transmission [14], the other does not account for outdoor biting and, like previous versions of this model [24], inaccurately treats diversion and mortality as independent, rather than sequentially coupled, events [53].

The specific results presented suggest that if high coverage levels can be achieved that are consistent with current World Health Organization targets [1,2], purely toxic products with no deterrence are predicted to generally provide superior protection to non-users and even users, especially where vectors feed exclusively on humans and a substantial amount of transmission occurs outdoors. Remarkably, this is even the case if that product confers no personal protection and only kills mosquitoes after they have fed. Products with purely mosquito-toxic profiles may be preferable to those with equivalent toxicity but which confer superior personal protection because of higher deterrence for programmes with universal coverage targets. Purely mosquito-toxic products which confer modest personal protection because they lack deterrence, or which confer none because they only kill mosquitoes after they have fed, will therefore require aggressive "catch up" campaigns to achieve high coverage rapidly and behaviour change communication strategies that emphasize the communal nature of protection.

As with all mathematical predictions, these predictions should only be considered as evidence of plausible hypotheses based on simplifying assumptions and imprecise parameterization. Lessons from learned from historic mistakes, specifically setting malaria prevention policy based on overconfident interpretation of malaria transmission models [3,4], are as relevant today as they ever were [54].

For example, one notable simplification to keep in mind is that complete gonotrophic concordance, meaning that each egg batch requires one and only one blood meal, has been assumed. In reality, the first blood meal typically requires at least one additional pre-gravid blood meal to achieve mature phase II development of the ovaries [55-57] and additional blood meals may even be taken during subsequent gonotrophic cycles [58]. While such increased feeding frequency would undoubtedly increase malaria transmission intensity in the absence of interventions such as LLINs or IRS, it would also be expected to increase the frequency of contact with such measures that mosquitoes would be exposed to early in their lives. Correspondingly, incorporating these subtle aspects of mosquito behaviour would most probably enhance the predicted impact of these measures upon transmission and therefore strengthen, rather than weaken, the contrasts between alternative target product profiles suggested here.

The only potentially major inaccuracy that seems obvious from the outputs of this model lies in the prediction that purely deterrent products will provide weak communal protection for non-users and may even increase their exposure. While this phenomenon appears plausible in theory and has been documented by field trials of some topical repellents [21], the experimental design of that study define situations in which only single users were protected, equivalent to negligible community level coverage (*C_h _*≈ 0) so community-level effects were neither realized nor evaluated. Furthermore, these predictions seem slightly at odds with observations from field trials of community-wide use of essentially untreated mosquito nets in both Tanzania [59] and Papua New Guinea [60]. In both cases, high coverage of nets lacking meaningful pesticidal properties but deterring mosquitoes through simple physical barrier effects successfully reduced malaria transmission. Combined with the anecdotal but reasonable attribution of reduced malaria transmission in many settings to housing improvements conferring similarly direct protection through similar physical barriers [61], these net trials suggest that the disappointing predictions for purely deterrent products described here should be interpreted with a degree of caution. The most obvious possible explanation for such possible discrepancies is the uncertainty associated with survival rate of mosquitoes foraging for blood or aquatic habitat and the extreme sensitivity of predictions to this parameter value and to baseline total availabilities of these resources [15,28,39,62-64]. To go beyond speculation based on sensitivity analysis of these critical but, as yet, unmeasured parameters, will clearly require the development of robust field methods, notably trapping of gravid *Anopheles *seeking oviposition sites [28].

With some notable exceptions, these simulations compare well with recent, less generalized, modelling analyses which examine choices between specific product types and combinations thereof [65,66]. Deliberately, no specific product has been named, nor has any measured parameter value for any specific product been set in any of these simulations. Instead, the product parameters have been tuned them across the full range of possible values so that ideal target product profiles can be objectively outlined for manufacturers and their clients to aim for prospectively rather than restrict discussion to the relative merits of currently available products and product combinations. Nevertheless, the parameter space explored here encompasses all the specific examples of product types evaluated in recent modelling analyses [65,66], resulting in predictions which are readily comparable in broad terms (Figures 3 and 6). Both these complementary recent studies [65,66] also conclude that IRS with a highly deterrent product such as DDT will have less impact than a predominantly insecticidal product such as IRS with bendiocarb or pyrethroid-based LLINs. However, their conclusions regarding combining such product types differ somewhat and the existing evidence base is insufficient to inform which of these three formulations appears most accurate. Chitnis *et al*. predict that supplementing a predominantly insecticidal LLIN products [65] with a highly deterrent one such as IRS with DDT will have a larger impact upon transmission than either one as a stand-alone measure. In contrast, the simulations of Yakob *et al. *[66], suggests the opposite: that placing a deterrent product in the same house as a predominantly insecticidal one will undermine the superior impact of the latter for exactly the reasons outlined here and captured in the convergence of outcomes with toxic and non-toxic products in Figure 3.

Perhaps the most important observation about the lack of consensus between these three model formulations is that sufficient field data do not exist to reliably compare them in terms of their predictive value. Recent reviews of the impact of IRS [10], and specifically IRS combined with insecticidal nets [10,67], both conclude that rigorous, large-scale, randomized controlled trials are conspicuous by their absence. An abundance of descriptive studies unambiguously demonstrate that IRS has massive overall impact and that combining with ITNs gives generally improved personal protection [10,49]. To the knowledge of the authors, however, no study yet exists in which the exclusively communal protection afforded to residents of unsprayed houses in IRS programmes has been measured as rigorously as it has for non-users of insecticidal nets in communities with high coverage levels [11,60,68-72]. Given these limitations in the evidence base for IRS as a stand-alone prevention strategy, it is perhaps unsurprising that the evidence base to support decisions about combining LLINs and IRS is insufficient and has become a common point of discussion for both theoreticians and practitioners [10,65-67].

Despite these limitations in both the consistency of outputs from alternative existing models and the empirical evidence base from the field, important lessons can be learned from these simulations which are intuitive and for which no caveats seem obvious. Although no evidence, based on rigorously randomized trials, for the *probability *of the deterrence-related attenuation of insecticidal impact have been reported, the existing descriptive evidence base presents a strong case for the *plausibility *[73] of this phenomenon.

*The effect of insecticidal attack was enhanced by the use of non-irritant insecticides *[49]

In fact, the ideal target product profile outlined here was already suggested during the previous malaria eradication era, when the impact of DDT which has a mixed deterrent-plus-toxic profile, was contrasted with that of Dieldrin which acts by contact toxicity only [13]:

*In many instances, Dieldrin proved to be more effective than DDT, but its higher cost, its toxicity to mammalians, and the fast-spreading resistance of A*[nopheles] *gambiae to this insecticide limited its further use in Africa *[49]

This model presented herein simply strengthens, explains and generalizes the plausibility of this argument, highlighting the lack of affordable, safe alternatives to Dieldrin with similarly non-deterrent properties. Three decades later, with insecticide resistance on the rise [74] and increasing levels of exophagy being reported for residual vector populations in Africa [26] and Asia [27], it is likely that several such active ingredients with distinct, complementary mechanisms will be required to prevent and manage insecticide resistance in the long term. These simulation results, therefore, serve as a timely reminder of the need for increased investment in development and evaluation of insecticidal products with purely toxic modes of action to achieve improved and sustained malaria vector control.

Even if the worst-case scenarios predicted here are confirmed through large-scale trials, it is important to remember that this analysis is restricted to typical LLIN or IRS products that are used indoors. One of the most interesting phenomena that this model captures, which is increasingly relevant as the importance of outdoor-biting vectors is recognized [26,27,54,75], is that the advantage of purely toxic products becomes greater where vector mosquitoes tend to feed outdoors (Figures 6 and 7). This suggests that deterrent activities can not only divert mosquitoes to animals or to humans lacking such products but also to the users themselves at times of the day when they are outside of the house and unprotected. This new insight arises directly and intuitively from the reformulation of how coverage and protection have been conceptualized and expressed mathematically. Further extensions of this approach may be useful for examining a wider diversity of possible pesticidal vector control products that target mosquitoes outside of houses [76-78] and even away from humans [24,79-81]. This conceptual and mathematical formulation represents a useful new tool for rational design of malaria vector control products. Furthermore, the way in which coverage and protection are conceptualized in this formulation represents a substantive change in thinking that may also enable more lucid re-examination of what these terms really mean in practice [82,83].

## Competing interests

The authors declare that they have no competing interests. The funders had no role in study design, data collection and analysis, decision to publish, or preparation of the manuscript.

## Authors' contributions

All authors formulated the research question and developed the conceptual basis of the model. GFK drafted the model formulation and manuscript in consultation with FOO, SJM and NC. The contents are the responsibility of GFK, NC, SJM and FOO and do not necessarily reflect the views of USAID, the United States Government or the Bill & Melinda Gates Foundation. All authors have read and approved the final version of the manuscript.
